# Digital Technologies: Advancing Individualized Treatments through Gene and Cell Therapies, Pharmacogenetics, and Disease Detection and Diagnostics

**DOI:** 10.3390/biomedicines10102445

**Published:** 2022-09-30

**Authors:** Peter R. Corridon, Xinyu Wang, Adeeba Shakeel, Vincent Chan

**Affiliations:** 1 Department of Immunology and Physiology, College of Medicine and Health Sciences, Khalifa University, Abu Dhabi P.O. Box 127788, United Arab Emirates; 2 Biomedical Engineering and Healthcare Engineering Innovation Center, Khalifa University, Abu Dhabi P.O. Box 127788, United Arab Emirates; 3 Center for Biotechnology, Khalifa University, Abu Dhabi P.O. Box 127788, United Arab Emirates

**Keywords:** digital technologies, cell therapies, pharmacogenetics, disease detection, diagnostics, personalized medicine

## Abstract

Digital technologies are shifting the paradigm of medicine in a way that will transform the healthcare industry. Conventional medical approaches focus on treating symptoms and ailments for large groups of people. These approaches can elicit differences in treatment responses and adverse reactions based on population variations, and are often incapable of treating the inherent pathophysiology of the medical conditions. Advances in genetics and engineering are improving healthcare via individualized treatments that include gene and cell therapies, pharmacogenetics, disease detection, and diagnostics. This paper highlights ways that artificial intelligence can help usher in an age of personalized medicine.

## 1. Introduction

The use of digital technologies to transform medicine through individualized treatments can revolutionize healthcare and well-being [1]. Such a paradigm shift can support our ability to meet the growing global demand for medical services [2]. Artificial intelligence (AI) is at the heart of this transformation and has been instrumental in deriving engineering solutions to monitor, process, and integrate large volumes of data at the population and individual levels [3]. Tailored treatments derived from AI will assist patients, physicians, and health systems in handling current challenges [4], as well as those of the future as patients age and diseases evolve [5].

Current medical practices generally focus on treating symptoms and ailments for large groups of people. Unfortunately, following these clinical practice guidelines can elicit differences in treatment responses, therapeutic effects, and adverse reactions based on genetic variations within various populations. Legacy delivery practices are often incapable of treating the underlying nature of a given condition and have produced a system with erratic quality and unsustainable costs [5]. Moreover, most clinical practice guidelines are oriented towards a single condition; conversely, patients often exhibit multimorbidities [6]. Thus, various treatment options are applied in parallel. Recent studies have shown that the synchronous application of independent clinical practices to manage multimorbidities is associated with adverse drug–drug or drug–disease responses [7], which is another factor driving the need to improve conventional medicine.

**Figure 1:**
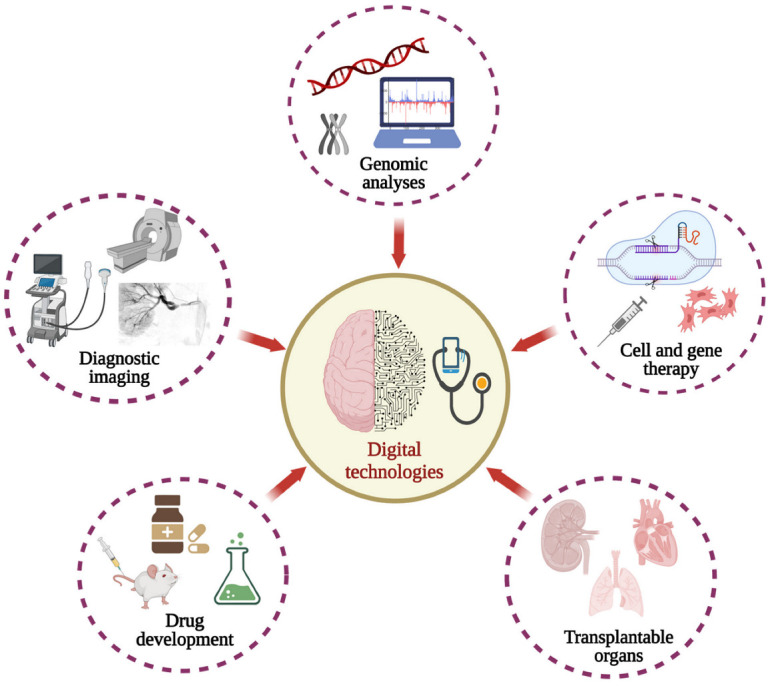
Illustration of digital technologies that are advancing individualized treatments.

Luckily, advances in genetics, engineering, and computational analyses have improved our understanding of the human body to redefine a path for healthcare. Specifically, this deeper understanding supports the development of treatments that will be far more tailored to individual needs. Emerging medical practices are focused on individual complexities that can manipulate disease interventions at the molecular level. DNA sequencing, high-throughput screening, molecular diagnostics, and advanced imaging methods embody some of the signs of progress of these emerging technologies and reveal interindividual diversity in unitary and multimodal disorders. This new era of modern medicine also produces ‘big data’, which requires colossal amounts of integration and analysis that are better suited for digital technologies [8]. To this end, significant research efforts are centered on regenerating diseased or lost tissues and organs, in-depth analyses extending beyond the clinician’s limits, and novel trends in disease prevention. This paper explores ways in which digital technologies will support a transition from conventional to personalized medicine by enhancing individualized treatments through the applications of gene and cell therapies, pharmacogenetics, and disease detection and diagnostics. Figure 1 is used to illustrate these concepts.

## 2. Gene and Cell Therapies

The completion of the Human Genome Project had a transformative effect on modern biomedical research and is a major factor supporting an age of personalized medicine [9]. Revising the previous outlook on genetics as a specialist’s interest, limited to addressing rare and life-threatening disorders, to a field that harnesses genetic information in all aspects of health care had a profound impact on medical doctrine [10,11]. This revised approach has increased our knowledge of the fundamental mechanisms involved in tissue/organ repair and identified promising options at the genetic and cellular levels [12]. 

Gene therapies are emerging to restore or counter malfunctioning genes in conditions adversely influencing a patient’s quality of life without mainstream pharmacological intervention, radiotherapy, or surgery [13]. This form of therapy has made significant progress since this concept arose in the 1960s and 1970s [14,15,16], but tragic failures in clinical settings [17], along with pervasive obstacles related to nucleic acid delivery [18,19,20,21,22,23], have limited its progression. However, a recent and significant achievement in this field has come from the CRISPR-Cas system. Using this technology, it became possible to elicit genetic modifications with greater precision for xenotransplantation by reducing the risk of rejection and transfer of zoonotic diseases [24]. Moreover, AI is poised to extend this gene-editing technology’s utility by predicting repair [25,26] and post-transplantation outcomes [27]. Other computational approaches are being developed to identify vectors for optimized gene delivery [28]. The evolving role of automation will also advance gene therapy by enhancing product quality and cost and time savings that can be translated to the clinics [29]. Nevertheless, simultaneous efforts must be made to enhance therapeutic delivery options, which invariably limit precision medicine applications [17,30]. 

Likewise, cell therapies rely on introducing exogenous cells to restore previously compromised or deteriorated tissues and organs. In practice, this technique entails transplanting human cells to repair or replace damaged structures. The cells may originate from the patient (autologous cells) or a donor (allogeneic cells). Compared to gene therapy, this system can be classified by its potential to regenerate and transform tissues/organs via different cell types such as stem or progenitor cells. In addition to using AI for production purposes, recent studies have found additional applications for digital technologies in this field of research. For instance, advanced computational models have collated millions of possible protein combinations into a catalog that could help target specific cell types in vivo [31] and provide predictions of the mortality risk associated with cell transplantation [32]. Digital technologies can also help determine cell viability, functionality, bioefficacy, and appropriate patient selection for cell therapy [33]. Another cell therapy platform is the organoid. Organoids are 3D multicellular tissue constructs that closely resemble functional organs, and their biological complexity provides new opportunities and challenges in data analytics [34], as well as chances to reduce the reliance on animal models [35], with higher physiological relevance [36] and automation [37].

## 3. Pharmacogenetics

Pharmacogenetics, also referred to as pharmacogenomics, focuses on how individuals respond to drug therapies based on their genetic makeup. This relatively new field relies on developing practical, safe medications and doses tailored to a person’s genetic makeup. The study of patient responses to specific drugs at the genome level can guide drug therapy evaluation; however, any variation to those genes can render a drug useless or cause adverse effects. Thus, the numerous factors that influence the response to specific treatment are worth noting. These factors may include, but are not limited to, age, body weight, sex, nutrition, infection history, organ function, supplement intake, and comedications. From a clinical perspective, pharmacogenetic practices can incorporate multiplexed data and help determine whether individual differences in genetic expression will affect drug metabolism and consequences on its therapeutic effect or toxicity [38].

Emerging digital applications can take advantage of rich multimodal data sets generated from various normal and pathological conditions to build a new generation of cost-effective and high-throughput screening tools that accurately unravel in vivo multiparametric states. For example, studies in this field have uncovered several inherited DNA variants that may cause hypersensitive states [39] or resistance to specific medications [40], making an otherwise safe therapy hazardous or ineffectual. Such compelling issues are driving scientists and drug developers to take a different approach. By conducting in vitro pharmacogenomic screenings, various gene-editing tools are helping us to uncover genomic modifications that can affect a drug’s clinical efficacy. Consequently, organ-on-a-chip technology is creating an alternative paradigm for toxicological assessments and preclinical drug development [41]. These multichannel 3D microfluidic-based cell culture systems form an integrated circuit simulating the activities, tissue mechanics, and physiological responses of an entire organ or an organ system, a type of artificial organ, and are again geared towards reducing the dependency on animals. Simultaneously, these approaches will dramatically accelerate the pace of these studies at a reduced cost [41]. Nevertheless, using genetic information to guide drug therapy requires rigorous training and manpower deployment, and, thus, automation can again be applied to help bring new drugs into clinical practice. 

## 4. Disease Detection and Diagnostics

Healthcare systems exist within dynamic environments in which clinicians are constantly challenged. The global shortages of medical practitioners [42,43] and diagnostics equipment [44] have put tremendous stress on already strained healthcare systems. Thus, there is an immediate need to increase practitioner and device pools and optimize their utilities. The COVID-19 pandemic has simultaneously exacerbated these issues and accelerated the pace of digital modalities to address these global problems. Studies within digital health have discovered new ways to use machine learning to detect and diagnose diseases, estimate patient prognosis and epidemic trends, and explore effective and safer drugs and vaccines [45]. More importantly, these automated practices can help existing systems better leverage healthcare resources. 

Several years ago, predictive modeling via multiple algorithms showed promise for early disease detection [46]. In more recent times, algorithm and computing qualities have improved, and it has been shown that the application of AI can significantly enhance diagnostic accuracies and efficiencies [47]. To illustrate this perspective, computational image analysis, and, thus, machine-learning-driven approaches, are especially poised to uncover new categories of biomarkers [48]. Traditionally, biomarkers have been classified by biological characteristics, such as a naturally occurring molecule or gene that objectively evaluates pathological and physiological processes [49]. The digital era aims to extend this definition with the imaging biomarker. An imaging biomarker is a biological characteristic that is detectable in an image. This characteristic is not a tool or a method but a measurable variable and indicator of normal or pathogenic conditions [48,50], that can rely on static image color, texture, and shape descriptors [50,51] or functional radiographic velocities and acceleration indices [52]. 

Imaging biomarkers can transform the role of conventional anatomical and functional imaging by redefining the detection and diagnostic processes on a decision-making level to identify the most appropriate procedure for optimizing individual care [48], thereby promoting precision medicinal practices. It is also important to note that many big data analytic systems have been criticized for failing to capture critical individual-level associations when combining data from large sets. It is thus critical to ensure that AI systems adapt to account for such issues to advance personalized treatment options for patients.

## 5. Conclusions

Conventional medical approaches focus on treating symptoms and ailments for large groups of people. Such approaches can generate differences in treatment responses, adverse reactions based on population variations, and may be incapable of treating the underlying pathophysiology of the condition. Furthermore, the dynamic healthcare environments, evolving nature of diseases, and the global shortage of medical equipment and practitioners highlight the substantial strain exerted on existing healthcare systems. New approaches are, thus, needed to address these needs, and we contend that digital technologies can individualize medical practice. This paradigm shift from conventional medicine to digital technologies can revolutionize healthcare by advancing individualized treatments via gene and cell therapies, pharmacogenetics, and disease detection and diagnostics.
